# Persistence of transferable oxazolidinone resistance genes in enterococcal isolates from a swine farm in China

**DOI:** 10.3389/fmicb.2022.1010513

**Published:** 2022-10-10

**Authors:** Zheren Huang, Yilin Bai, Qin Wang, Xue Yang, Tiejun Zhang, Xuan Chen, Hongning Wang

**Affiliations:** ^1^Animal Disease Prevention and Food Safety Key Laboratory of Sichuan Province, College of Life Sciences, Sichuan University, Chengdu, China; ^2^College of Veterinary Medicine, Northwest A&F University, Yanglin, China

**Keywords:** Enterococci, oxazolidinone, *poxtA2-cfr(D)* co-harboring, optrA, poxtA, genomic analyses, phylogenetic analysis, genetic environment

## Abstract

The appearance of transferable oxazolidinone resistance genes poses a major challenge to public health and environmental safety. These genes not only lead pathogenic bacteria to become resistant to linezolid but also reduce sensitivity to florfenicol, which is widely used in the veterinary field. To verify the dissemination of oxazolidinone resistance genes in enterococcal isolates from pigs at different production stages in a swine farm in China, we collected 355 enterococcal isolates that were resistant to florfenicol from 600 (150 per stage) fresh fecal swabs collected from a swine farm. Through initial PCR screening and whole-genome sequencing, 175 isolates harboring different oxazolidinone resistance genes were identified. All isolates carried the *optrA* gene. A total of 161 (92%, 161/175) isolates carried only the *optrA* gene. Three (1.71%, 3/175) isolates carried both the *optrA* and *poxtA* genes, and 11 (3.1%, 11/175) isolates contained the *optrA* gene and *poxtA2* and *cfr(D)* variants. A total of 175 isolates that harbored oxazolidinone resistance genes included 161 *E. faecalis*, 6 *E. faecium*, and 8 *E. hirae*. By sequencing the whole genomes, we found that the 161 isolates of *E. faecalis* belonged to 28 different STs, including 8 new STs, and the 6 isolates of *E. faecium* belonged to four different STs, including one new ST. The phylogenetic tree based on SNPs of the core genome showed that both clonal spread and horizontal transfer mediated the diffusion of oxazolidone resistance genes in enterococcal isolates at specific stages in pig farms. Moreover, enterococcal isolates carrying oxazolidone resistance genes could spread from breeding pigs to fattening pigs, while transferable oxazolidone resistance genes in enterococcal isolates could persist on a pig farm throughout all production stages. Representative enterococcal isolates with different oxazolidinone resistance genes were further studied through Nanopore sequencing. We identified a novel plasmid, pM4-80 L4 (15,008 bp), carrying the *poxtA2* and *cfr(D)* genes in enterococcal isolates at different stages. We also found three different plasmids harboring the *poxtA* gene with high genetic variation, and all *poxtA* genes were flanked by two copies of IS*1216E* elements. In addition, four genetically distinct plasmids carrying the *optrA* gene were identified, and Tn*554* was found to mediate chromosome-localized *optrA* gene transfer. Our study highlighted that transferable oxazolidinone resistance genes in enterococcal isolates could persist throughout all production stages on a pig farm, and the prevalence and dissemination of oxazolidinone resistance genes in enterococcal isolates from animal farms should be continually monitored.

## Introduction

Gram-positive enterococcal bacteria occur widely in the intestines of humans and animals. Enterococci in animals used for food production may contaminate food and the environment, creating a risk of human infection through anthropozoonosis (He et al., 2016). *Enterococcus faecalis* and *Enterococcus faecium* often cause urinary tract and soft tissue infections, and in severe situations, they may cause septicemia or meningitis (Yi et al., 2022). Notably, *E. faecium* and *E. faecalis* are important nosocomial pathogens worldwide (Bender et al., 2018). It has been reported that enterococci can not only exhibit inherent resistance to some antibiotics, but also easily acquire new resistance genes located on mobile genetic elements from other sources, including other bacteria. Thus, multidrug resistance can arise readily in enterococci, resulting in limited clinical treatment options (He et al., 2016). In addition, vancomycin-resistant *E. faecium* (VRE) has been listed by the World Health Organization as a pathogen requiring high vigilance since it was discovered at the end of the last century (WHO, 2017). Oxazolidinone antibiotics, mainly linezolid and tedizolid, have a bactericidal effect on various gram-positive bacteria (Wilson et al., 2008; Moellering, 2014). In 2000, the US FDA introduced linezolid for clinical treatment. Linezolid is usually used to treat severe clinical infections [Methicillin-resistant Staphylococcus aureus (MRSA) and Vancomycin-resistant Enterococcus faecium (VRE)] and is known as the last line of defense for the treatment of gram-positive bacteria (Bender et al., 2018). With the extensive clinical use of linezolid in the clinic, linezolid-resistant enterococci have gradually emerged. The emergence of linezolid-resistant enterococci poses a great challenge to human clinical treatment and public health (Schwarz et al., 2021).

Linezolid resistance mechanisms are associated with ribosomal mutations in the 23S rRNA and/or L3, L4 and L22 ribosomal proteins (Wang et al., 2020). However, the appearance of transferable oxazolidinone-resistant determinants in enterococci or other bacteria in many regions of the world should not be ignored (Ewa, 2018). *Cfr* is the first reported transferable oxazolidinone resistance gene and encodes a 23S rRNA methyltransferase. Bacteria carrying the c*fr* gene exhibit resistance to phenicols, oxazolidinones, lincosamides, pleuromutilins, and streptogramin A (PhLOPSA phenotype; Long et al., 2006; Shen et al., 2013). Since the *cfr* gene was originally discovered, four *cfr*-like genes have been reported worldwide, namely, *cfr(B)*, *cfr(C)*, *cfr(D)* and *cfr (E)* (Deshpande et al., 2015; Zhang et al., 2017; Stanley et al., 2019). *OptrA* is the second oxazolidinone resistance gene, reported in 2015, and encodes a ribosomal protection protein of the ABC-F family. Bacteria carrying the *optrA* gene exhibit resistance to oxazolidinones and phenicols (Wang et al., 2015). Since the initial description of the *optrA* gene, it and its many variants have been reported in several countries, revealing that it is more difficult to treat oxazolidinone-resistant bacteria (Schwarz et al., 2021). Unfortunately, another ribosomal protection protein of the ABC-F family, *poxtA*, was reported in MRSA of clinical origin in 2018 (Antonelli et al., 2018; Crowe-McAuliffe et al., 2022). The *poxtA2* variant was also reported in 2021 (Baccani et al., 2021). Now, *poxtA* has been detected in clinical samples, animal samples and even marine plankton samples in many countries (Papagiannitsis et al., 2019; Dejoies et al., 2021). Although oxazolidinone antibiotics have never been approved for use in livestock breeding, we often detect the transferable determinants of oxazolidinone resistance in enterococci isolated from animals used in food production (Wang et al., 2020). Florfenicol is a phenicol compound that has broad spectrum antibacterial activity and few side effects and is used to treat respiratory and intestinal bacterial infections in animals used in food production (Crowe-McAuliffe et al., 2022). It was reported that the abundance of oxazolidinone resistance genes in livestock feces was related to florfenicol residue (Kim, 2021). Given that phenicol resistance could be caused by the extensive use of florfenicol, the widespread use of florfenicol in the veterinary field will not only promote the spread of antibiotic resistance genes of florfenicol but also promote the spread of oxazolidone resistance genes, which will cause great public health concerns.

Transferable oxazolidinone resistance genes are common in enterococci isolated from pig farms, as has been reported worldwide recently (Hao et al., 2019; Lei et al., 2019, 2021; Fioriti et al., 2021). However, there are few reports on the dissemination mode of transferable oxazolidinone resistance genes in pigs at different production stages in large-scale pig farms. In this study, we investigated the presence of oxazolidinone resistance genes in florfenicol-resistant enterococci isolated from a large-scale swine farm in China and determined the dissemination mechanisms of oxazolidinone resistance genes in enterococci isolated from healthy pigs in different production stages.

## Materials and methods

### Sample collection and florfenicol-resistant enterococcal isolation

The large-scale swine farm (approximately 1,000 breeding pigs and 10,000 fattening pigs) is located in Sichuan, one of China’s major pig raising provinces. In October and November 2021, Fresh fecal swabs were taken from 150 breeding pigs in the breeding pig section of the farm and from 450 fattening pigs aged between approximately 2, 4, and 6 months (150 fattening pigs each stage) in the fattening pig section of the farm. The swab samples were inoculated into 3 ml of Buffer Peptone Water (Oxoid, Basingstoke, United Kingdom) containing 8 mg/l florfenicol. The culture was incubated overnight at 37°C and 180 RPM, and following incubation, 0.1 ml aliquots of culture were streaked onto Pfizer Enterococcus Selective Agar (Qingdao Hope Bio-Technology, China) supplemented with florfenicol (8 mg/l), and the selective plate was incubated overnight at 37°C. If the plate had colonies, we randomly selected one enterococcal colony from the plate. Then, we used an automated system (BD Diagnostic Systems, Sparks, MD, United States) to identify the enterococcal isolates.

### Detection of oxazolidinone resistance genes and antimicrobial susceptibility testing

Initial PCR screening was performed for all florfenicol-resistant enterococcal isolates using generic primers directed against the *optrA*, *cfr* and *poxtA* genes (Bender et al., 2019), and variants of *optrA*, *cfr* and *poxtA* were then identified by whole-genome sequencing. We sequenced the positive PCR products with Sanger (Chengdu Sangon, China). According to Clinical and Laboratory Standards Institute (CLSI) guidelines (Clinical and Laboratory Standards Institute, 2020), the minimum inhibitory concentrations (MICs) of linezolid, tedizolid, florfenicol, vancomycin, ampicillin, doxycycline, chloramphenicol and tetracycline were determined by the broth microdilution method. *E. faecium* ATCC29212 was used as a quality control strain for antimicrobial susceptibility testing.

### Whole-genome sequencing and genomic analyses of enterococcal isolates that harbored oxazolidinone resistance genes

The MiniBEST bacterial genomic DNA Extraction Kit (Takara, Dalian, China) was used to extract the genomic DNA of enterococcal isolates containing oxazolidone resistance genes (*optrA*, *poxtA* and *cfr*). The entire genomes were sequenced using the Illumina HiSeq platform (150-bp paired-end reads, with an average coverage of approximately 200-fold). We used SPAdes_3.13.0 software to collect original sequencing data to map the genomes. Antimicrobial resistance genes were identified by using ResFinder 4.1.^1^ Linezolid resistance determinants were searched by LRE-Finder 1.0.^2^

To investigate the genetic environments of different oxazolidinone resistance genes, we selected 12 enterococcal isolates for further research, and the basis of our selection is described below. First, among the 11 enterococcal isolates carrying the *optrA* gene and the *poxtA2* and *cfr(D)* variants, there were three ST-type isolates, and we selected one isolate for each ST (M2-9, M4-54, and M4-80). Second, we selected all enterococcal isolates carrying the *optrA* and *poxtA* genes, for a total of three isolates (B6, B54, and M2-95). Third, because there have been many reports on the genetic environment of *optrA* gene, and our manuscript mainly wanted to focus on the genetic environment of *poxtA* gene, *poxtA2* and *cfr(D)* variants, we selected four representative enterococcal isolates out of 161 enterococcal isolates carrying only the *optrA* gene (M6-97, M6-130, B83, and B126). Finally, to study the phenomenon of heteroresistance, we selected two enterococcal isolates carrying only the *optrA* gene (M2-77 and M2-82). A Nanopore MinION Rapid Sequencing Kit was used to further sequence the genomes of representative enterococcal isolates with different oxazolidinone resistance genes. Using Nanopore sequencing data combined with Illumina sequencing data, the complete genome sequences were assembled by Unicycler. PCR linkage confirmed that there was a circular plasmid containing a transferable oxazolidinone resistance gene. Easyfig v2.2.2^3^ was used for comparative analyses of the plasmids.

### Molecular typing and phylogenetic analysis of enterococcal isolates that harbored oxazolidinone resistance genes

Sequence types (STs) were assigned on the basis of conventional MLST loci. STs of enterococcal isolates were determined by PubMLST,^4^ and the new STs were also assigned by PubMLST. MLST-based minimum spanning trees were created by GrapeTree v1.3.2.^5^ The core genome is present in all individuals of the same species, and the genes in the core genome are generally related to the stable biological function and phenotypic characteristics of the species, most of which are housekeeping genes (Tettelin et al., 2005; Yang and Gao, 2022). Using the core genomes of enterococci to construct phylogenetic trees can reduce the influence of variable genomes on genetic phylogenetic relationships. Sequences of isolates that harbored oxazolidinone resistance genes were annotated using Prokka v1.12 software, and the core genome was identified using Roary v3.11.2, then the SNPs of the core genome were extracted by Harvest tools (Seemann, 2014; Page et al., 2015; Dejoies et al., 2021). A phylogeny based on SNPs of the core genome was constructed by FastTree v2.1.11.^6^ The Newick file for the phylogenetic tree was modified in iTOL.

### Transfer experiments

Using the criteria described previously in “Whole-genome sequencing and genomic analyses of enterococcal isolates that harbored oxazolidinone resistance genes,” eight isolates (M4-80, B6, B54, M2-95, M2-77, M2-82, M6-97, and M6-130) carrying different plasmids that were representative of the 175 enterococcal isolates carrying the oxazolidinone resistance gene in this study were selected as donors. The rifampicin-resistant *E. faecalis* JH2-2 was used as the recipient. Conjugal transfer was performed on a filter membrane as described previously (Brenciani et al., 2016). The donor and recipient bacteria were mixed in a 1:1 ratio on the filter membrane. Transconjugants were selected on Brain Heart Infusion Agar (Oxoid, Basingstoke, United Kingdom) plates containing 2 mg/l linezolid and 25 mg/l rifampicin. The transfer frequency was expressed as the ratio of the cell number (cfu/ml) of the transconjugant to that of the recipient.

Transconjugants were evaluated for their susceptibility to linezolid, tedizolid, chloramphenicol, florfenicol, tetracycline and doxycycline. Then, the detection of transferable oxazolidinone resistance genes, 16S rDNA sequencing and whole genome sequencing were also used to confirm transconjugants.

### Nucleotide sequence accession numbers

The genomes of enterococcal isolates that harbored oxazolidinone resistance genes in this study have been deposited in the National Center for Biotechnology Information and registered BioProject number PRJNA856057. The complete nucleotide sequences of nine plasmids harboring different transferable oxazolidinone resistance genes have been deposited in GenBank and assigned accession numbers OP046170–OP046178.

## Results

### Prevalence of oxazolidinone resistance genes in florfenicol-resistant enterococcal isolates

In this study, 355 isolates of florfenicol-resistant enterococcal isolates were collected from 600 fresh fecal swabs taken from pigs at different stages from breeding pig section and fattening pig section of the pig farm. Through initial PCR screening and whole-genome sequencing, 175 isolates harboring different oxazolidinone resistance genes were identified (Table 1). All isolates carried the *optrA* gene. A total of 161 (92%, 161/175) isolates carried only the *optrA* gene. Three (1.71%, 3/175) isolates carried both the *optrA* and *poxtA* genes, and eleven (3.1%, 11/175) isolates contained the *optrA* gene and *poxtA2* and *cfr(D)* variants (Table 1). A total of 175 isolates that harbored oxazolidinone resistance genes included 161 *E. faecalis*, 6 *E. faecium*, and 8 *E. hirae* (Table 1). With respect to the isolation rate of enterococci carrying oxazolidinone resistance genes in each production stages, 49 (32.67%, 49/150) isolates came from the breeding pig section of the pig farm. The remaining isolates came from the fattening pig section of the pig farm, with 47 (31.33%, 47/150) from 2-month fattening pigs, 40 (26.67%, 40/150) from 4-month fattening pigs, and 37 (24.67%, 37/150) from 6-month fattening pigs.

**Table 1:** Enterococcal isolates that harbored oxazolidinone resistance genes in this study.

Species	MLST	Numbers	Acquired resistance genes		MICs (mg/l)^a^							
			Oxazolidinoe	Phenicol	LZD	TZD	VA	FFC	DOX	CHL	TET	PEN
*faecalis* (*n* = 161)	16	4	*optrA*	*fexA*	4	0.5	0.5	128	16	32	128	1
	19	1	*optrA*	*fexA*	8	1	1	128	16	64	256	2
	21	7	*optrA*	*fexA*	8	1	2	128	8	64	128	0.5
	59	6	*optrA*	*fexA*	4	0.25	0.5	128	16	64	128	0.25
	86	1	*optrA*	*fexA*	8	0.5	2	64	16	128	256	0.5
	227	12	*optrA*	*fexA*	4	0.25	0.5	128	16	64	128	0.5
	256	9	*optrA、poxtA2、cfr(D)*	*fexA*	8	2	1	128	16	128	256	1
	314	15	*optrA*	*fexA*	4	0.25	0.5	128	32	256	256	1
	444	1	*optrA*	*fexA*	4	0.5	1	128	32	256	256	1
	476	2	*optrA*	*fexA*	4	1	1	128	16	64	256	0.5
	480	1	*optrA*	*fexA*	4	0.5	0.5	128	32	256	256	1
	506	40	*optrA*	*fexA*	4	0.25	0.5	128	16	64	128	0.5
	535	3	*optrA*	*fexA*	4	0.5	0.5	128	32	256	256	1
	591	1	*optrA*	*fexA*	4	0.5	1	128	32	64	64	0.5
	631	2	*optrA*	*fexA*	4	1	1	16	16	64	256	1
	632	8	*optrA*	*fexA*	4	0.5	1	64	8	64	64	0.5
	634	7	*optrA*	*fexA*	8	1	1	16	16	64	256	1
	868	8	*optrA*	*fexA*	4	0.5	0.5	128	32	256	256	1
	902	18	*optrA*	*fexA*	8	1	2	256	16	256	256	0.5
	982	5	*optrA*	*fexA*	4	0.5	0.5	128	4	32	128	1
	**1,250**	3	*optrA*	*fexA*	4	1	2	128	16	256	256	0.5
	**1,251**	1	*optrA、poxtA2、cfr(D)*	*fexA*	8	2	1	128	16	128	128	1
	**1,252**	1	*optrA*	*fexA*	4	0.5	1	128	32	64	64	0.5
	**1,253**	1	*optrA*	*fexA*	4	1	1	16	16	64	256	1
	**1,254**	6	*optrA*	*fexA*	8	1	2	128	16	64	128	1
	**1,255**	1	*optrA、poxtA2、cfr(D)*	*fexA*	8	2	1	128	16	128	128	1
	**1,256**	1	*optrA*	*fexA*	8	1	2	128	16	64	128	0.5
	**1,257**	4	*optrA*	*fexA、fexB*	4	0.125	2	256	16	256	256	0.5
*faecium* (n = 6)	184	1	*optrA*	*fexA*	4	0.125	0.5	256	16	64	256	16
	323	1	*optrA、poxtA*	*fexA*	16	1	0.25	128	128	16	32	128
	1,630	1	*optrA、poxtA*	*fexA、fexB*	8	1	0.5	256	32	256	256	32
	**2,165**	3	*optrA*	*fexA*	4	1	0.5	256	16	64	128	2
E*. hirae* (*n* = 1)	–	1	*optrA、poxtA*	*fexA*	16	1	0.25	128	16	64	128	8
*E. hirae* (*n* = 7)	–	7	*optrA*	*fexA*	4	1	0.5	256	16	64	128	2

a LZD, linezolid; TZD, tedizolid; VAN, vancomycin; FFC, florfenicol; DOX, doxycycline; CHL, chloramphenicol; TET, tetracycline; PEN, penicillin.

Antimicrobial susceptibility testing indicated that the MIC values of linezolid against 161 enterococcal isolates that harbored only the *optrA* gene ranged from 4 to 8 mg/l, and the MIC values of tedizolid ranged from 0.125 to 1 mg/l (Table 1). Three isolates that carried the *optrA* and *poxtA* genes were resistant to linezolid and tedizolid. The MICs of the three isolates to linezolid and tedizolid were 8–16 mg/l and 1 mg/l, respectively (Table 1). In addition, 11 isolates carrying both the *optrA* gene and the *poxtA2* and *cfr(D)* variants showed resistance to linezolid and tedizolid, and the MICs for these two antibiotics were 8 and 2 mg/l, respectively (Table 1). A total of 175 isolates exhibited a multidrug resistance phenotype and were resistant to chloramphenicol, florfenicol, tetracycline and doxycycline. Moreover, three isolates were resistant to penicillin (MICs ≥ 16 mg/l).

### Genomic analyses of enterococcal isolates that carried oxazolidinone resistance genes

By sequencing the whole genomes of 175 isolates that carried oxazolidinone resistance genes, we found that the *cfr* and *poxtA* genes carried by 11 *E. faecalis* isolates were the *cfr(D)* and *poxtA2* variants. The homology of the *cfr(D)* gene and wild type (accession number NG_067192) was 99.9%. The *poxtA2* variant was recently detected in two *E. faecalis* isolates and one *E. casseliflavus* isolate from manure of a swine farm in Italy (Baccani et al., 2021). Unlike *poxtA*, the *poxtA2* variant (accession number MZ171245) was not truncated by an *IS*1216 insertion at the 3′ end; thus, a new sequence consisting of eight amino acids (TPEEEQKY) replaced the six amino acids (GSVAKF) of the wild-type protein. Six different *optrA* variants were found by alignment of the *optrA* amino acid sequences with those found in *E. faecalis* E349 (Supplementary Table 1; Wang et al., 2015). Five of these variants have been reported previously (Cai et al., 2018). One variant, DKD (G40D, I287K, G393D), has not been reported previously (Supplementary Table 1). Different linezolid MICs were also present in isolates harboring different *optrA* variants (Supplementary Table 1).

In addition, all 175 isolates that contained the phenicol resistance gene *fexA* and five isolates also carried the *fexB* gene. All the isolates carried the tetracycline resistance genes *tet(L)* and/or *tet(M)* and macrolide resistance genes *erm(A)* and/or *erm(B).* Five isolates carried the macrolide resistance gene *msr(C)*, and the M2-95 strain harbored both the mosaic tetracycline resistance gene *tet(S/M)* and the macrolide resistance gene *msr(C).* Four isolates of *E. faecalis* of ST1257 contained the mosaic tetracycline resistance gene *tet(O/W/32/O)* and chloramphenicol resistance gene *cat.* Thirteen to nineteen mutations in the *pbp5* gene were found in six isolates of *E. faecium*. Two isolates carried the *clpL* gene.

### Molecular typing and phylogenetic analysis of enterococcal isolates that harbored oxazolidinone resistance genes

**Figure 1:**
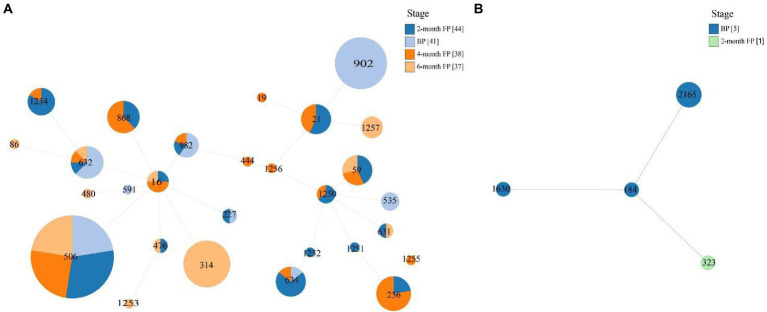
Multilocus Sequence Typing (MLST)-based minimum spanning trees of *E. faecalis* **(A)** isolates and *E. faecium* **(B)** isolates. The stage is indicated by different colors.

**Figure 2:**
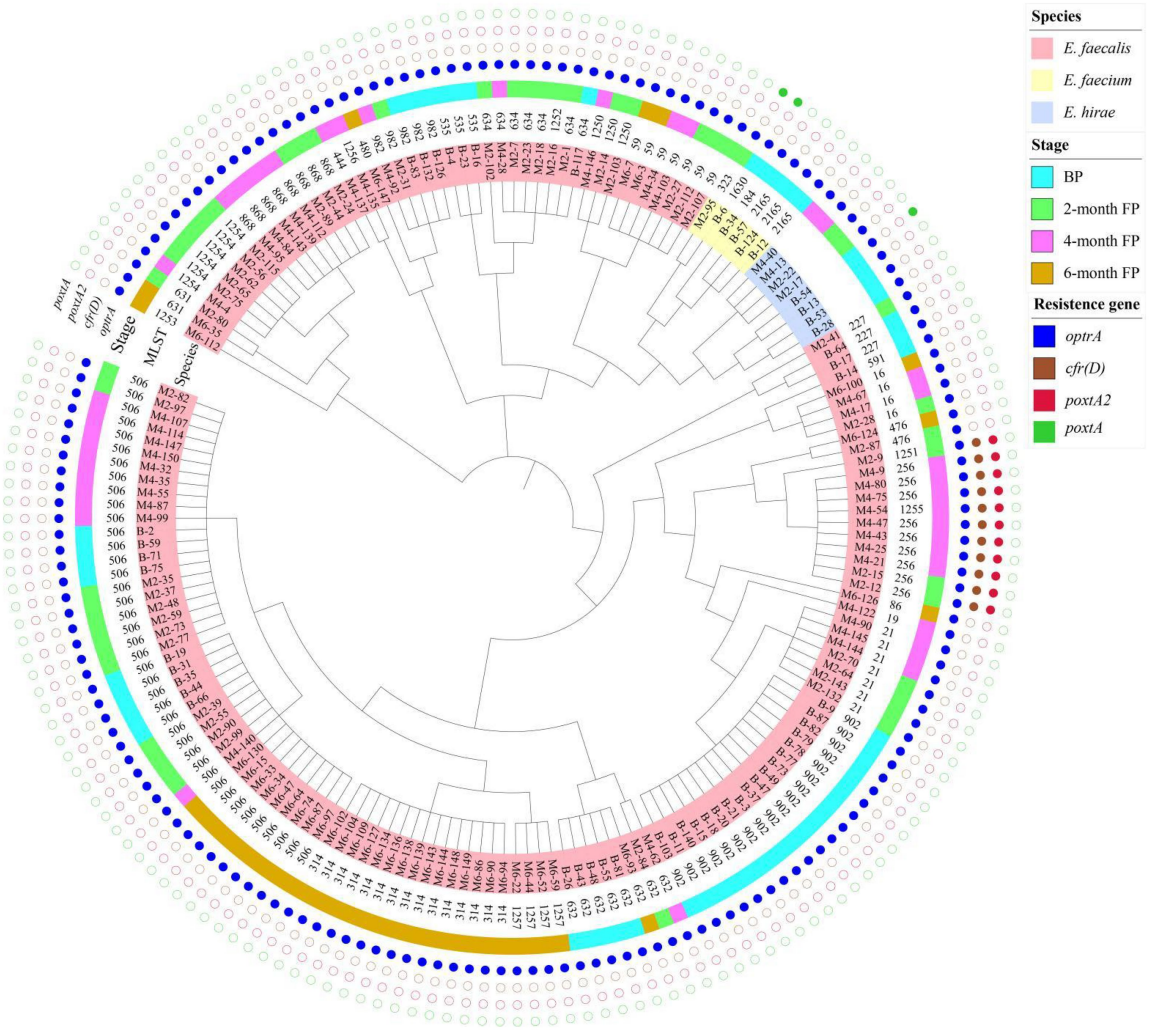
Phylogenetic tree based on SNPs of the core genome for 175 enterococcal isolates that harbored different oxazolidinone resistance genes. The species, stage, and oxazolidinone resistance genes are indicated by different colors.

MLST showed that the 161 isolates of *E. faecalis* that harbored oxazolidinone resistance genes belonged to 28 different STs, including ST21, ST59, ST256, ST314, ST506, ST632, ST634, ST868, ST902, and ST982, and eight new STs were assigned by PubMLST, including ST1250, ST1251, ST1252, ST1253, ST1254, ST1255, ST1256, and ST1257 (Table 1). Six *E. faecium* isolates that harbored oxazolidinone resistance genes belonged to 4 different STs, including ST184, ST323, ST1630 and one new ST, ST2165, assigned by PubMLST (Table 1). The 161 *E. faecalis* isolates were widely isolated from pigs in different production stages (Figure 1A). It is worth noting that isolates ST506 and ST632 were found in the samples from all four stages (Figure 1A). However, the distribution of the six *E. faecium* isolates was relatively homogeneous across different stages (Figure 1B). A phylogenetic tree based on SNPs of the core genome showed that the 175 enterococcal isolates carrying oxazolidone resistance genes were divided into 35 branches (Figure 2). The isolates with the same ST type were clustered into the same branch in the phylogenetic tree (Figure 2). For the isolates isolated from a certain stage, all ST902 isolates isolated from breeding pigs belonged to one branch, and the ST21 isolates isolated from 2-month fattening pigs also clustered together in the phylogenetic tree. Similar phenomena were found in 4-month fattening pigs and 6-month fattening pigs (Figure 2). For the isolates isolated from different stages, ST506 isolates carrying the *optrA* gene belonged to one clade and were isolated from samples in all four production stages (Figure 2). ST632 isolates carrying the *optrA* gene were clustered in the phylogenetic tree, and they were prevalent in all four production stages (Figure 2). The *poxtA* gene was carried by two isolates of *E. faecium* and one strain of *E. hirae*, and the *poxtA2* and *cfr(D)* variants were carried by ST256, ST1251 and ST1255 isolates of *E. faecalis* (Figure 2).

### The genetic environments of different oxazolidinone resistance genes

**Figure 3:**
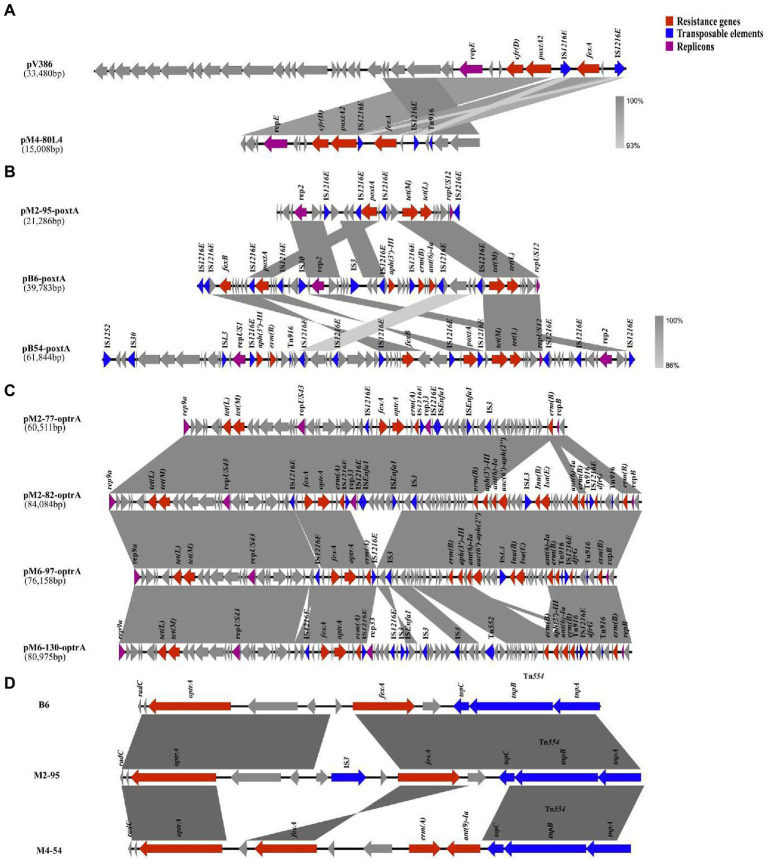
The genetic environments of different oxazolidinone resistance genes. Genes and ORFs are shown as arrows, and their transcription orientations are indicated by the arrowheads. **(A)** The genetic structure of pM4-80L4. **(B)** The genetic structures of three poxtA-carrying plasmids. **(C)** The genetic structures of four optrA-carrying plasmids. **(D)** The genetic structures of three different chromosomal *optrA* gene clusters.

The genetic structures of plasmids containing oxazolidinone resistance genes and the genetic environments of different oxazolidinone resistance genes are shown in Figure 3. In three *E. faecalis* isolates (M2-9, M4-54 and M4-80) that carried the *optrA* gene and the *poxtA2* and *cfr(D)* variants, bioinformatic data revealed that the *optrA* gene of three isolates was localized to the chromosome at *Tn*554, and a novel plasmid carrying *poxtA2* and *cfr(D)* variants, named pM4-80 L4, was found concomitantly in these three isolates. This 150,008 bp plasmid (35.5% GC content) contained 17 ORFs. BLASTN analysis revealed that pM4-80 L4 was 98.15% identical (coverage 99%) to pV386 (33,48 kb in size; accession number MZ603802.1) carrying *cfr(D)* and *poxtA2* variants. pV386 was recently found in an *E. faecalis* strain that was isolated from a swine environment in central Italy (Cinthi et al., 2022). Similar to pV386, pM4-80 L4 showed that the *poxtA2* gene was closely associated with the *cfr(D)* gene (Figure 3A). Upstream of the *poxtA2* gene, the *fexA* gene was found to be surrounded by two copies IS*1216E* in the same direction, and the rep*1* gene (belonging to the Inc18 family) was also detected downstream of the *cfr(D)* gene (Figure 3A).

Three isolates (B6, B54, and M2-95) harbored both the *optrA* and *poxtA* genes. In these three isolates, we found that the *optrA* gene was localized on the chromosome, whereas the *poxtA* gene was located in a plasmid in all isolates. Three different *poxtA*-carrying plasmids with sizes ranging from 21.2 to 61.8 kb were obtained after hybrid assembly (Table 2). Analysis of the *poxtA* nucleotide sequence location in each isolate showed that they exhibited 100% identity to that of *S. aureus* AOUC-0915, in which *poxtA* was initially described (Antonelli et al., 2018). Every *poxtA* gene was flanked by IS*1216E* at the left and/or right ends (Figure 3B). Two *poxtA*-carrying plasmids from two *E. faecium* isolates (B6 and M2-95) belonged to the same type that harbored rep*2* and rep*US12* replicons (Figure 3B). Two plasmids exhibited high genetic variation, and both of them carried the tetracycline resistance genes *tet(L)* and *tet(M)*. pB6-poxtA also carried the phenicol resistance gene *fexB*, macrolide resistance gene *erm(B)* and aminoglycoside resistance gene *aph(3′)-III*; IS*3* and IS*30* were also found in pB6-poxtA (Figure 3B). The final *poxtA*-carrying plasmid, pB54-poxtA, from the *E. hirae* strain (B-54), carried three replicons (rep*2*, rep*US12*, and rep*US1*) and harbored the *fexB*, *tet(L)*, *tet(M)*, *aph(3′)-III* and *erm(B)* resistance genes, and IS*1252*, IS*L3*, IS*30*, and Tn*916* were also identified in pB54-poxtA (Figure 3B).

**Table 2:** Information of the different plasmids harboring transferable oxazolidinone resistance genes and antimicrobial susceptibilities of the transconjugants and recipient isolates.

Transconjugant^a^	Frequency	MICs (mg/l)^b^					Plasmids				Resistance genes
		LZD	TZD	CHL	FFC	TET	DOX	Name	Size (bp)	Replications	
M4-80^T1^	7.6 × 10^−7^	8	1	128	64	128	16	pM4-80 L4	15,008	rep*E*	*poxtA2*, *cfr(D)*, *fexA*
M4-80^T2^	8.2 × 10^−7^	2	0.25	8	4	1	2	pM4-80 L3	57,887	rep*A*,rep*B*	*erm(B)*, *aph(3′)-III*, *dfrG*
B6^T^	3.9 × 10^−7^	8	0.5	64	128	128	32	pB6-poxtA	39,783	rep*2*, rep*US12*	*poxtA*, *aph(3′)-III*, *ant(6)-Ia*, *fexB*, *erm(B)*, *tet(M)*, *tet(L)*
B54^T^	2.5 × 10^−7^	8	0.5	128	128	128	64	pB54-poxtA	61,844	rep*US1*, *rep2,* rep*US12*	*poxtA*, *aph(3′)-III*, *fexB*, *erm(B)*, *tet(M)*, *tet(L)*
M2-95^T^	3.2 × 10^−7^	4	0.25	64	64	128	32	pM2-95-poxtA	22,186	rep*2*, rep*US12*	*poxtA*, *tet(M)*, *tet(L)*
M2-77^T^	7.4 × 10^−6^	4	0.25	64	64	64	16	pM2-77-optrA	60,511	rep*9a*, *rep33*, rep*B*, rep*US43*	*optrA*, *fexA*, *erm(A), erm(B)*, *tet(M)*, *tet(L)*
M2-82^T^	4.3 × 10^−6^	4	0.25	64	64	64	16	pM2-82-optrA	84,084	rep*9a*, *rep33*, rep*B*, rep*US43*	*optrA*, *aph(3′)-III*, *ant(6)-Ia*, *aac(6′)-aph(2″)*, *fexA*, *erm(A), erm(B)*, *tet(M)*, *tet(L), lsa(E), lnu(B), dfrG*
M6-97^T^	4.4 × 10^−7^	4	0.5	64	128	128	32	pM6-97-optrA	76,158	rep*9a*, *repB*, rep*US43*	*optrA*, *aph(3′)-III*, *ant(6)-Ia*, *aac(6′)-aph(2″)*, *fexA*, *erm(A), erm(B)*, *tet(M)*, *tet(L), lsa(E), lnu(B), dfrG*
M6-130^T^	7.1 × 10^−6^	4	0.5	64	128	128	32	pM6-130-optrA	89,075	rep*9a*, *rep33*, rep*B*, rep*US43*	*optrA*, *aph(3′)-III*, *ant(6)-Ia*, *fexA*, *erm(A), erm(B)*, *tet(M)*, *tet(L), dfrG*
*E. faecalis* JH2–2	–	1	0.25	4	2	0.125	0.125	–	–	–	–

a T, transconjugant.

b LZD, linezolid; TZD, tedizolid; CHL, chloramphenicol; FFC, florfenicol; TET, tetracycline; and DOX, doxycycline.

Among the 161 enterococcal isolates that carried only the *optrA* gene, 27 isolates had the *optrA* gene localized to the chromosome at *Tn*554, while 134 isolates had the *optrA* gene localized to a plasmid. From the four *optrA*-carrying isolates that we selected (M2-77, M2-82, M6-97, and M6-130), four different *optrA*-carrying plasmids were identified, three of which belonged to the same type and carried rep*9a,* rep*US43*, rep*33* and rep*B* replicons (Table 2). The remaining plasmid carried the rep*9a,* rep*US43* and rep*B* replicons (Table 2). The genetic structure of the *optrA-*carrying plasmids is shown in Figure 3C. All *optrA* genes were associated with the phenicol resistance gene *fexA* and the macrolide resistance gene *erm(A)*, and a similar genetic context IS*1216E*–*fexA*–*optrA*–*erm(A)*–IS*1216E* was found in four different *optrA*-carrying plasmids (Figure 3C). In the two *optrA*-carrying isolates we selected (B83 and B126) and the previous six isolates with *optrA* gene localization on chromosomes (M2-9, M4-54, M4-80, B6, B54, and M2-95), four different chromosomal *optrA* gene clusters were found. Three of these *optrA* genes were associated with Tn*554* inserted into the *radC* gene, and the *fexA*—*optrA* segment was detected upstream of the Tn*554* transposon (Figure 3D). The M4-54 isolate also carried the macrolide resistance gene *erm(B)* and the aminoglycoside resistance gene *ant(9)-Ia* upstream of the *fexA*-*optrA* segment, and IS*3* inserted into the *fexA*-*optrA* segment was also found in M2-95 (Figure 3D). The genetic context IS*1216E*–*fexA*–*optrA*–*erm(A)*–IS*1216E* on the chromosome was identified in the remaining isolate (B54; Supplementary Figure 1).

Interestingly, the clonally related isolates had the heteroresistant genotype in ST506 and ST256, specifically in the ST506 isolates M2-77 and M2-82 (genotype M2-77 lacked *aph(3′)-III*, *aac(6′)-aph(2″)*, *ant(6)-Ia, lsa(E)*, *lnu(B)* and *dfrG* genes compared with M2-82) and the ST256 isolates M4-75 and M4-80 (genotype M4-75 lacked *aph(3′)-III, erm(B)* and *dfrG* genes compared with M4-80). Bioinformatics’ results revealed the genetic environments of differential resistance genes in clonally related isolates. Compared with M2-77, IS*1216E* and Tn*916* might mediate the insertion of a 21,511 bp fragment that contained differential resistance genes into plasmids (pM2-82) of M2-82 (Figure 3C). However, compared with M4-75, M4-80 probably received a complete plasmid (pM4-80L3, 57.9 kb in size) that harbored differential resistance genes from the external environment (Supplementary Figure 1).

### Transferability of plasmids containing different transferable oxazolidinone resistance genes

Using antimicrobial susceptibility testing, the detection of transferable oxazolidinone resistance genes, 16S rDNA sequencing and whole-genome sequencing, nine transconjugants containing transferable oxazolidinone resistance genes were obtained, with frequencies ranging from 7.1 × 10^−6^ to 2.5 × 10^−7^ (Table 2). The MICs of linezolid against those transconjugants varied from 4 to 8 mg/l, and the MICs of tedizolid varied from 0.25 to 1 mg/l (Table 2).

## Discussion

Knowledge of the distribution of antimicrobial-resistant isolates in the food chain and edible animals is important in determining the potential risk of antimicrobial-resistant isolates to human health (Yoon et al., 2020). Although oxazolidinones have been approved for human use only, the *cfr, optrA* and *poxtA* genes have been detected in enterococcal isolates of animal and environmental origin and recently were even found in enterococci from coastal seawater samples (Freitas et al., 2021; Schwarz et al., 2021). Enterococci in edible animals that carry oxazolidinone resistance genes poses a threat to public health and the surrounding environment. In this study, we investigated the prevalence and genetic characteristics of oxazolidinone resistance genes in enterococcal isolates to better understand their resistance profiles and the dissemination of oxazolidinone resistance genes in enterococcal isolates obtained from pigs at different stages on a swine farm.

Since the three oxazolidinone resistance genes were discovered, these genes have been detected in enterococcal isolates from hospital patients, as well as livestock in the community and veterinary hospital (Carmen et al., 2018). In this study, we reported the presence of *optrA*, *poxtA*, *poxtA2* and *cfr(D)* in 49.3% (175/355), 1.71% (3/175), 3.1% (11/355) and 3.1% (11/355) of florfenicol-resistant enterococcal isolates, respectively. The detection rate of the *optrA* gene was higher than that reported by Wang et al., who found that 24.8% (37/149) of enterococcal isolates of swine origin harbored the *optrA* gene (Wang et al., 2015). The prevalence of the *poxtA* gene was lower than that observed by Hao et al., who reported that the *poxtA* gene was present in 57.9% (66/114) of the florfenicol-resistant enterococcal isolates from two swine farms in Henan Province in China (Hao et al., 2019). The prevalence of the *poxtA* gene was close to that in florfenicol-resistant enterococci of swine origin in Italy (4.14%, 6/145; Fioriti et al., 2020). Moreover, Cinthi et al. reported that two *E. faecalis* isolates and one *E. casseliflavus* isolate from a pig farm environment carried *poxtA2* and *cfr(D)* variants (Cinthi et al., 2022). These results indicated that the prevalence of three oxazolidinone resistance genes in enterococcal isolates from different swine farms exhibited huge differences, which might be associated with different veterinary antimicrobial agent usage schemes.

This study confirmed that WGS played an indispensable role in understanding the resistance profiles of oxazolidinone resistance genes and monitoring the dissemination of oxazolidinone resistance genes in enterococcal isolates. Searching for resistance genes among 355 florfenicol-resistant enterococcal isolates indicated no mutations in 23S rRNA (G2576T and G2505A) or in the L3, L4 and L22 ribosomal proteins in 175 enterococcal isolates carrying oxazolidinone resistance genes (Baccani et al., 2021); the remaining 180 florfenicol-resistant enterococcal isolates were not resistant to oxazolidinone, and no chromosomal mutations associated with oxazolidinone resistance were detected. In this study, three enterococcal isolates carried two oxazolidinone resistance genes, and 11 isolates carried three oxazolidinone resistance genes. Eleven *E. faecalis* harbored both the *cfr*-like variant *cfr(D)* and the *poxtA2* variant. The *poxtA2* variant was first identified from an isolate of *E. gallinarum* from a healthy child in Bolivia (Baccani et al., 2021). We also found six *optrA* variants, one of which has not been reported in *E. faecalis*. The presence of multiple oxazolidinone resistance genes in one strain might enhance resistance to oxazolidinones, and different variants of different oxazolidinone resistance genes and other factors in the different enterococcal species contribute to the level of linezolid resistance, which requires further investigation (Cai et al., 2018). We also found other antibiotic resistance genes in these 175 enterococcal isolates that harbored resistance genes for oxazolidines, such as tetracycline (*tet(M)*, *tet(S/M)* and *tet(O/W/32/O)*), and macrolides (*erm(A)*, *erm(B)* and *msr(C)*). Two isolates also carried the *clpL* gene, which encodes a chaperone family protein of HSP100/Clp (caseinolytic protease). It was found mainly in gram-positive bacteria, which might be associated with decreased penicillin susceptibility (Tran et al., 2011). Although we did not find vancomycin resistance genes, more attention should be given to monitoring the VRE that carries transferable oxazolidinone resistance genes (Yi et al. 2022). A total of 175 enterococcal isolates harbored multiple resistance genes, which indicated a broad antibiotic resistance spectrum of enterococcal isolates. This might make it difficult to treat antibiotic-resistant enterococcal isolates on swine farms.

On the basis of conventional MLST loci, we classified 175 enterococcal isolates harboring oxazolidinone resistance genes. A total of 161 *E. faecalis* isolates belonging to 28 different STs, including eight new STs, were identified. Of those clones, the most prevalent was ST506 (24.84%, 40/161). ST902 (11.18%, 18/161) was the second most prevalent clone. Moreover, four different STs and one new ST were found in six *E. faecium* isolates. Using WGS, MLST-based minimum spanning trees of *E. faecalis* isolates (161) and *E. faecium* isolates (6) were constructed. We found that the STs of *E. faecalis* were distributed across multiple production stages. In particular, ST506 and ST632 isolates were found in pigs at four production stages, indicating that these ST isolates may spread in pigs of different stages on swine farms. However, the distribution of *E. faecium* (6) isolates was simple because the number of *E. faecium* isolates was relatively small. A phylogenetic tree of the 175 enterococcal isolates was constructed based on SNPs of the core genome. The isolates with the same STs were clustered into the same branch on the phylogenetic tree, suggesting that there was a clonal correlation among the isolates. This result was consistent with the MLST results. For the isolates isolated from a certain stage, 18 isolates of ST902 from breeding pigs showed high similarity, and four isolates of ST21 from 2-month fattening pigs also showed high similarity. In contrast, different ST isolates isolated from the same production stage showed high diversity and did not cluster together in the phylogenetic tree. These results suggest that both clonal spread and horizontal transfer might mediate the diffusion of oxazolidone resistance genes in enterococcal isolates at specific stages in pig farms. For the isolates isolated from different production stages, we found that ST506 and ST632 isolates isolated from four stages showed high similarity, and the same ST isolates were also found to have clonal correlation between two adjacent stages (BP to 2M-FP, 2M-FP to 4M-FP, 4M-FP to 6M-FP). This meant that enterococcal isolates carrying oxazolidone resistance genes could spread from breeding pigs to fattening pigs, while transferable oxazolidone resistance genes in enterococcal isolates could persist throughout all production stages on a pig farm. Finally, the *poxtA2* and *cfr(D)* variants were always carried by ST256 isolates isolated from the 2-month fattening pigs and 4-month fattening pigs. In addition, ST1251 and ST1255 isolates isolated from the 2-month fattening pigs and 4-month fattening pigs also carried the *poxtA2* and *cfr(D)* variants, which were also close to ST256 isolates in the phylogenetic tree. This result indicated that ST1251 and ST1255 may have evolved from ST256.

Representative enterococcal isolates carrying different types of oxazolidinone resistance genes were further sequenced to investigate the locations and genetic environments of different oxazolidinone resistance genes. Mobile genetic elements contribute significantly to the transmission of resistance genes. For the 11 isolates carrying the *optrA* gene and the *poxtA2* and *cfr(D)* variants, we identified a novel plasmid, named pM4-80 L4, that was prevalent in different STs of *E. faecalis* isolates from 2-month fattening pigs and 4-month fattening pigs. The plasmid harboring the *poxtA2* and *cfr(D)* variants was similar to pV386 (accession number MZ603802.1), an isolate of swine origin from Italy, indicating that these two plasmids might have the same origin (Cinthi et al., 2022). The emergence of pM4-80 L4 harboring the *poxtA2* and *cfr(D)* variants demonstrates that intense genetic exchanges between enterococcal isolates promoted the spread of oxazolidinone resistance determinants.

The *poxtA* gene shares 32% homology with the *optrA* gene (Dejoies et al., 2021). To date, there is no report on *poxtA* in enterococcal chromosomes. Among three enterococcal isolates carrying the *optrA* and *poxtA* genes, we identified three different plasmids carrying the *poxtA* gene. All *poxtA* genes were flanked by two IS*1216E* element copies in the same direction, which was consistent with other reports. The IS*1216E*—*poxtA*—IS*1216E* segment in our study is similar to that found in *S. aureus* AOUC-0915 and clinical *E. faecium* isolates from Italy and France, indicating that the genetic background of *poxtA* is relatively unique (Bender et al., 2018; Carmen et al., 2018; Dejoies et al., 2021; Coccitto et al., 2022). Moreover, a variety of antibiotic resistance genes were found on these plasmids, revealing co-transmission of antibiotic resistance genes. The main differences among insert sequences (ISs) are that they have different transposase properties and catalyze different chemical reactions (Wilson et al., 2008). The three different plasmids exhibited high genetic variation. Three to eight copies of IS*1216E* were found in the three plasmids, and we also identified other types of ISs. Different ISs might promote the diversity of *poxtA*-harboring plasmids. Early studies suggested that the transposition of ISs in the genome was random, while recent experiments confirmed that ISs are more inclined to insert into the plasmid in bacteria, which is conducive to the spread of ISs with plasmids as vectors (Siguier et al., 2006). Therefore, more attention should be given to bacterial antibiotic resistance mediated by ISs.

We also described the environment of *optrA* genes in different locations in this study. Because there have been many reports on the genetic environment of *optrA* gene, and our manuscript mainly wanted to focus on the genetic environment of *poxtA* gene, *poxtA2* and *cfr(D)* variants, we selected four representative isolates from 161 isolates carried only *optrA* gene (M6-97 and M6-130 represent isolates with *optrA* gene localized to a plasmid, B83 and B126 represent isolates with *optrA* gene localized to the chromosome). We obtained two *optrA-*carrying plasmids by hybrid assembly in two isolates (M6-97 and M6-130). By comparing the sequences of the two *optrA-*carrying plasmids with the sequences of 134 isolates in which the *optrA* gene was localized to the plasmid, we found that regardless of the plasmid, its sequence coverage with the sequence of 134 isolates reached more than 85%. This showed that the *optrA-*carrying plasmids among 134 isolates were similar to these two plasmids obtained by hybrid assembly. In our study, we selected four isolates carrying only the *optrA* gene (M2-77, M2-82, M6-97, and M6-130), and four genetically distinct *optrA-*carrying plasmids were identified. A similar genetic context, IS*1216E*–*fexA*–*optrA*–*erm(A)*–IS*1216E*, was found in four plasmids, which was consistent with *E. faecalis* from human and animal origin (He et al., 2016). This indicates that IS*1216E* might promote the cotransfer of *optrA*, *fexA* and *erm(A)* among plasmids. In summary, IS*1216E* in Gram-positive bacteria (*Enterococcus*, *Streptococcus suis* and *Listeria monocytogenes*) belongs to the IS*6*/IS*26* family of bacterial ISs and plays an important role in mobilizing antimicrobial resistance genes (Shan et al., 2020). Tn*554* mediation of *optrA* gene transfer was identified in the chromosome carrying the *optrA* gene, consistent with other reports (He et al., 2016; Kang et al., 2019). The *optrA* gene was flanked by transposon (Tn*554*) or insertion sequences (IS*1216E*), indicating that the *optrA* gene can be transferred among different bacterial genera and species.

In addition, the phenomenon of heteroresistance complicates the analysis of antibiotic resistance in bacteria. We found heteroresistance of clonally related isolates in ST506 and ST256 by genomic analyses and phylogenetic tree construction. Then, we preliminarily clarified the mechanism of heteroresistance emergence, which was likely that mobile genetic elements mediated the insertion of DNA fragments or the acquisition of external plasmids. Last, but not least, these results suggest that caution must be taken to avoid the dissemination of oxazolidinone resistance genes in the environment as they reveal the genetic environment of different oxazolidinone resistance genes.

## Conclusion

Our study highlighted that transferable oxazolidinone resistance genes in enterococcal isolates could persist throughout all production stages on a pig farm. Different mobile genetic elements, such as plasmids (pM4-80 L4), IS*1216E* and Tn*554*, mediated the dissemination of oxazolidinone resistance genes in enterococcal isolates in the swine farm. These results indicate that oxazolidinone resistance genes in enterococcal isolates had diverse dissemination characteristics in different production stages of large-scale pig farms. Although few data show that oxazolidinone-resistant enterococci could be directly transmitted from animals to humans, enterococci in animals used for food production could be an important repository of transferable oxazolidinone resistance genes. The prevalence and dissemination of oxazolidinone resistance genes in enterococcal isolates from animal farms should be continually monitored.

## Data availability statement

The data presented in this study are deposited in the National Center for Biotechnology Information. The complete nucleotide sequences of nine plasmids harboring different transferable oxazolidinone resistance genes have been deposited in GenBank and assigned accession numbers OP046170–OP046178.

## Author contributions

HW and ZH designed the study and supervised the work. YB, QW, XY, TZ, and XC participated, coordinated, and analyzed the data. ZH and YB wrote the manuscript. All authors contributed to the article and approved the submitted version.

## Funding

This work was supported by the National Natural Science Foundation of China (grant no. U21A20257).

## Conflict of interest

The authors declare that the research was conducted in the absence of any commercial or financial relationships that could be construed as a potential conflict of interest.

## Publisher’s note

All claims expressed in this article are solely those of the authors and do not necessarily represent those of their affiliated organizations, or those of the publisher, the editors and the reviewers. Any product that may be evaluated in this article, or claim that may be made by its manufacturer, is not guaranteed or endorsed by the publisher.

## Footnotes

^1^ https://cge.cbs.dtu.dk/services/ResFinder/

^2^ https://cge.cbs.dtu.dk/services/LRE-finder/

^3^ http://mjsull.github.io/Easyfig/

^4^ https://pubmlst.org/

^5^ https://pypi.org/project/grapetree/1.3.2/

^6^ http://www.microbesonline.org/fasttree/
