# Supplementary material for: Persistence of transferable oxazolidinone resistance genes in enterococcal isolates from a swine farm in China
Source: Front Microbiol. 2022 Oct 10;13:1010513. doi: 10.3389/fmicb.2022.1010513 (PMC9589348; doi:10.3389/fmicb.2022.1010513)
Supplement: Supplementary file 1 [file Table_1.docx]

Supplementary Material

**Supplementary Table 1.** *OptrA* variants in enterococcal isolates characterised in this study and their MIC of Linezolid.

| Strain | OptrA | | | MICs (mg/L) |
| --- | --- | --- | --- | --- |
|  | Variant | Alterations | Location(kb) | Linezolid |
| 1. *hire* B-54 | KLDP | T112K, S147L, Y176D, T481P | Chromosome | 16 |
| *E. faecium* B-6 | DD | Y176D, G393D | Chromosome | 8 |
| *E. faecails* B-83 | Wild-type | none | Chromosome | 4 |
| *E. faecails* B-126 | Wild-type | none | Chromosome | 4 |
| *E. faecails* M2-77 | RDK | I104R, Y176D, E256K | Plasmid (60.5kb) | 4 |
| *E. faecails* M2-82 | RDK | I104R, Y176D, E256K | Plasmid (84kb) | 4 |
| *E. faecails* M2-95 | D | Y176D | Chromosome | 16 |
| *E. faecails* M2-9 | DKD | G40D, I287K, G393D | Chromosome | 8 |
| *E. faecails* M4-54 | DKD | G40D, I287K, G393D | Chromosome | 8 |
| *E. faecails* M4-80 | DKD | G40D, I287K, G393D | Chromosome | 8 |
| *E. faecails* M6-97 | RDK | I104R, Y176D, E256K | Plasmid (76.1kb) | 4 |
| *E. faecails* M6-130 | RDK | I104R, Y176D, E256K | Plasmid (80.9kb) | 4 |
