# Supplementary material for: Persistence of transferable oxazolidinone resistance genes in enterococcal isolates from a swine farm in China
Source: Front Microbiol. 2022 Oct 10;13:1010513. doi: 10.3389/fmicb.2022.1010513 (PMC9589348; doi:10.3389/fmicb.2022.1010513)
Supplement: Supplementary file 2 [file Image_1.pdf]

Supplementary Material

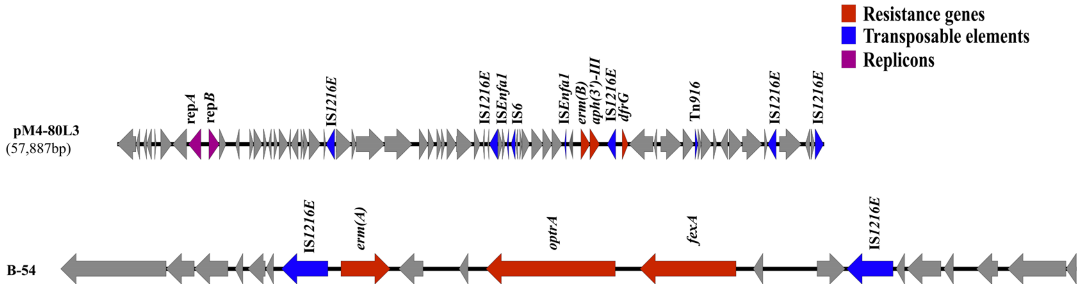

Supplementary Figure 1. Genetic structures of another plasmid of M4-80 and chromosomal optrA gene cluster of B-54. Genes and ORFs are shown as arrows, and their orientations of transcription are indicated by the arrowheads. Replicons, antimicrobial resistance genes and transposase elements are in purple, red and blue, respectively.
